# Supplementary figures and images for: Ratiometric quorum sensing governs the trade-off between bacterial vertical and horizontal antibiotic resistance propagation
Source: PLoS Biol. 2020 Aug 14;18(8):e3000814. doi: 10.1371/journal.pbio.3000814 (PMC7449403; doi:10.1371/journal.pbio.3000814)

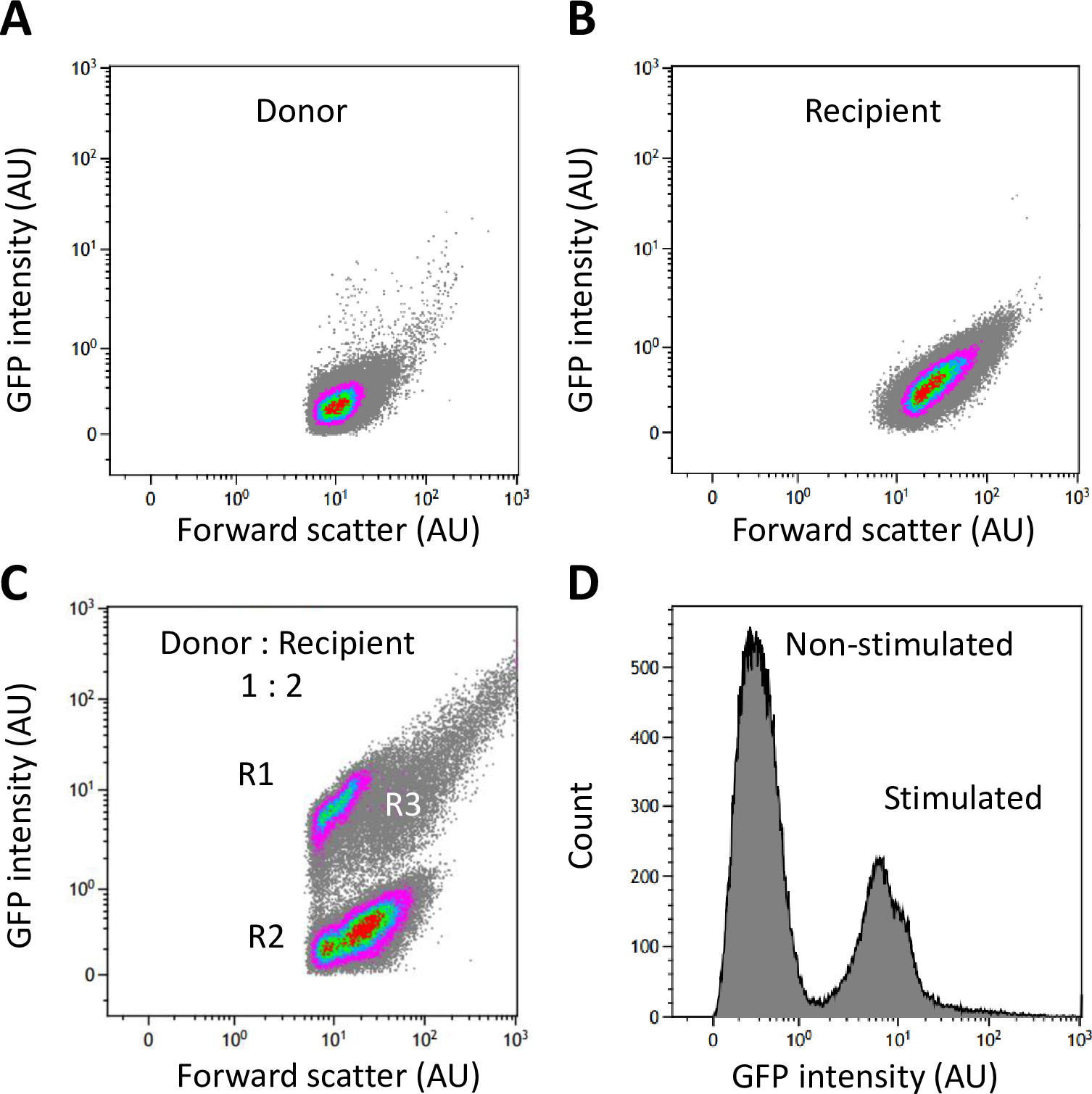

Supplement: S1 Fig — (A, B) Scatter plot of pure donor OG1RF(pCF10-GFP) (A) and recipient OG1SSp (B) cell populations. (C) Example of a coincubation reaction of donors and recipients at a recipient-biased ratio. The mean single-cell intensity of donors was obtained from population R1. Population R2 corresponds to a mixture of autofluorescent donors and nonactivated recipients. Population R3 corresponds to donor–recipient pairs (and higher-order aggregates, i.e., events in R3 with higher fluorescence values than events in R1 [“tail”] were not considered in the analysis). To estimate the fraction of activated donors (Fig 2C), we considered the three populations and the known experimentally determined initial ratio in the calculation (see Coincubation experiments). (D) Histogram with GFP intensities from the data in (C). The underlying numerical data are shown in S6, S7 and S8 Data. GFP, green fluorescent protein. (TIF) [file pbio.3000814.s001.tif]

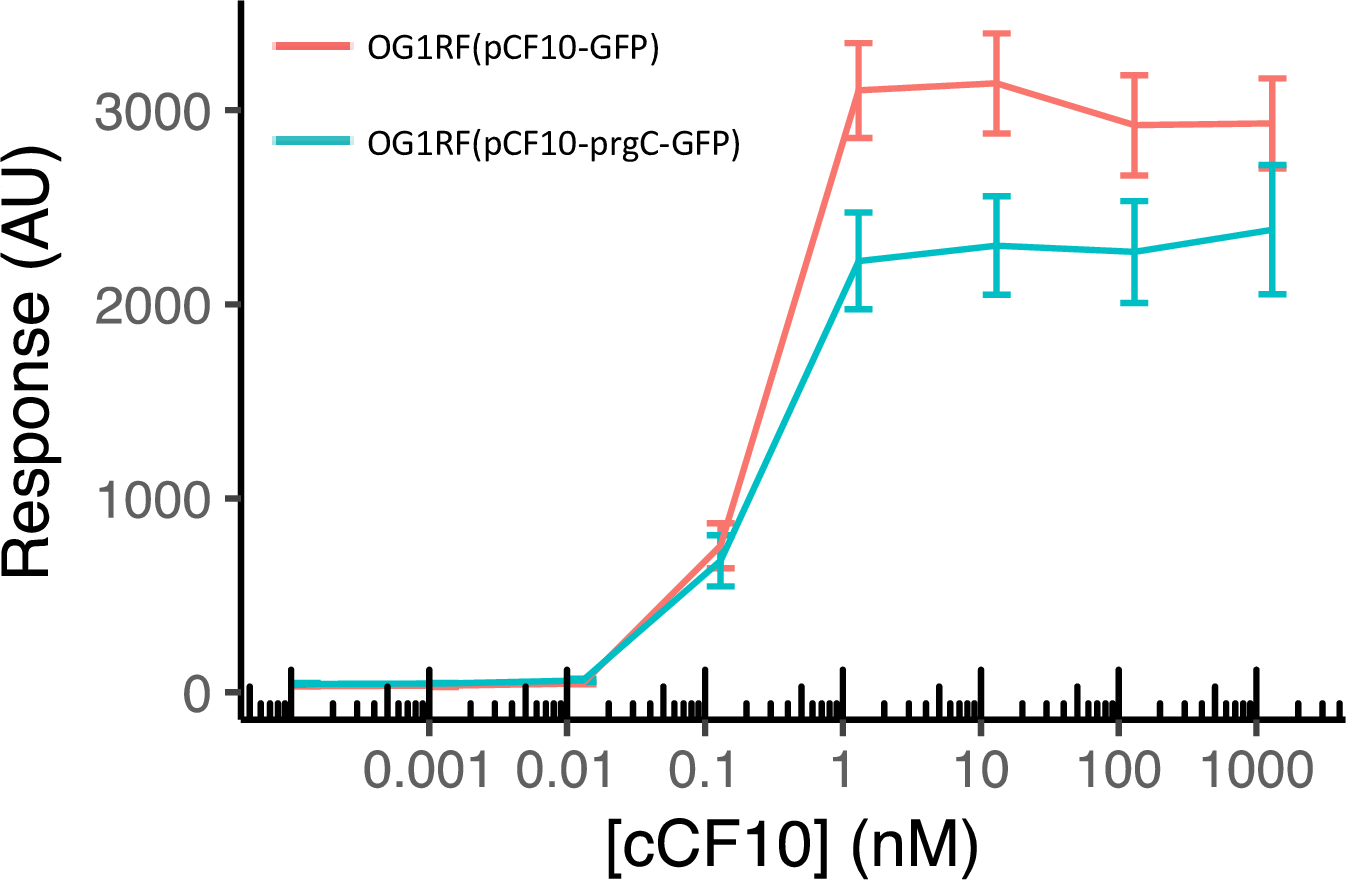

Supplement: S2 Fig — Comparison of (OG1RF(pCF10-GFP)) (ΔprgU, increased output) and wild-type (OG1RF(pCF10-prgC-GFP)) cCF10 dose-responses. Error bars are SEM (n = 4). The underlying numerical data are shown in S9 Data. GFP, green fluorescent protein. prg, pheromone responding gene. (TIF) [file pbio.3000814.s002.tif]

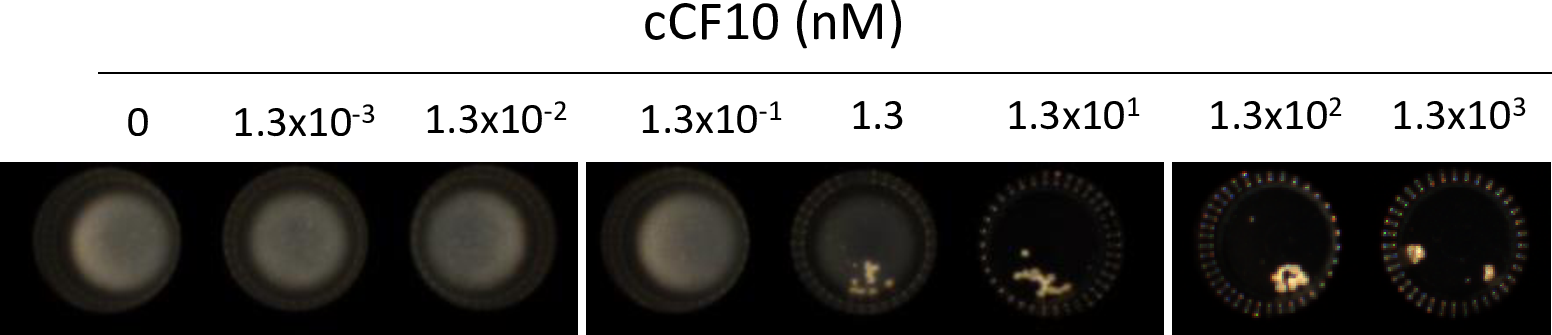

Supplement: S3 Fig — Orbital shaking–dependent macroscopic self-aggregate formation in OG1RF(pCF10) as a function of cCF10 concentration. The physiological (heterophilic) range of pheromone concentration required to induce detectable Asc10 expression lies below 1 nM cCF10. (TIF) [file pbio.3000814.s003.tif]

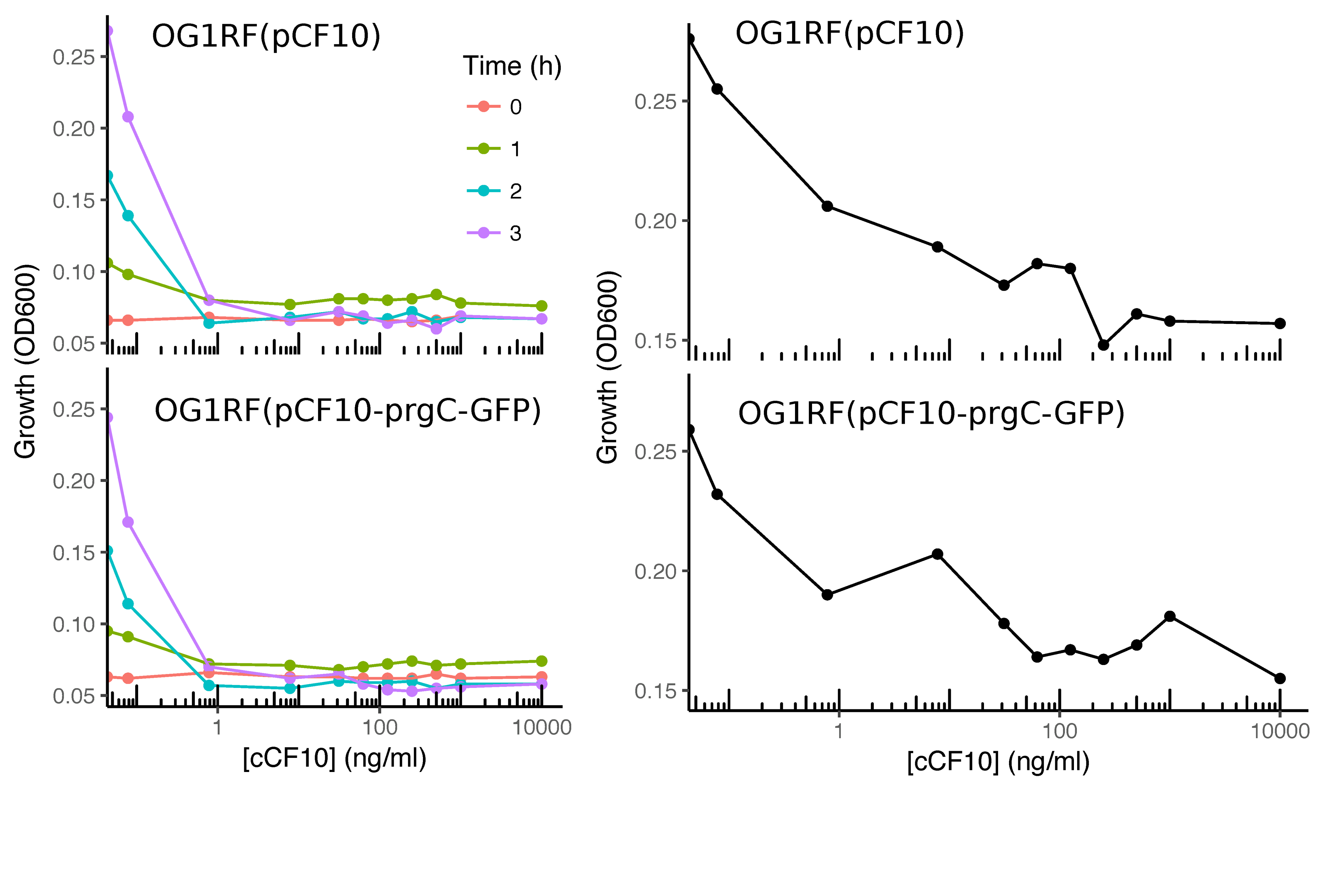

Supplement: S4 Fig — (A, B) WT (OG1RF(pCF10)) versus OG1RF(pCF10-prgC-GFP) reporter comparison in an aggregate formation assay (A) and postaggregate dispersion cell density measurement (B) (see Aggregation and turbidity assay). Note the increase in turbidity at 1 hour in (A), the time at which aggregation brings it back to the baseline. The underlying numerical data are shown in S10 Data. GFP, green fluorescent protein; prg, pheromone responding gene; WT, wild-type. (TIF) [file pbio.3000814.s004.tif]

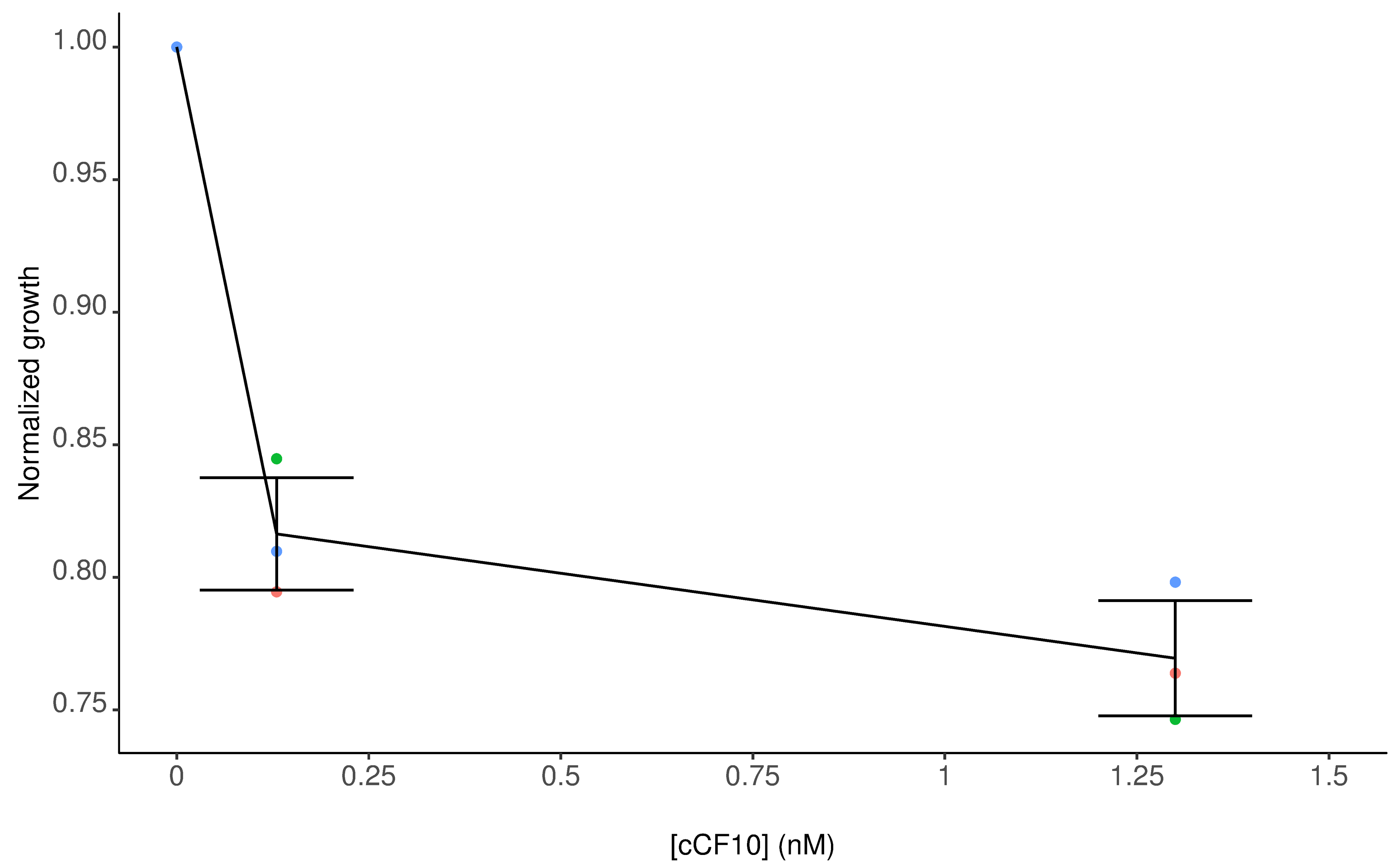

Supplement: S5 Fig — Growth reduction in the physiological (sub-nanomolar) response range in growth/adhesion assays (see Adherence/growth assay). Error bars are SD of n = 3 independent experiments (colors). The underlying numerical data are shown in S11 Data. (TIF) [file pbio.3000814.s005.tif]

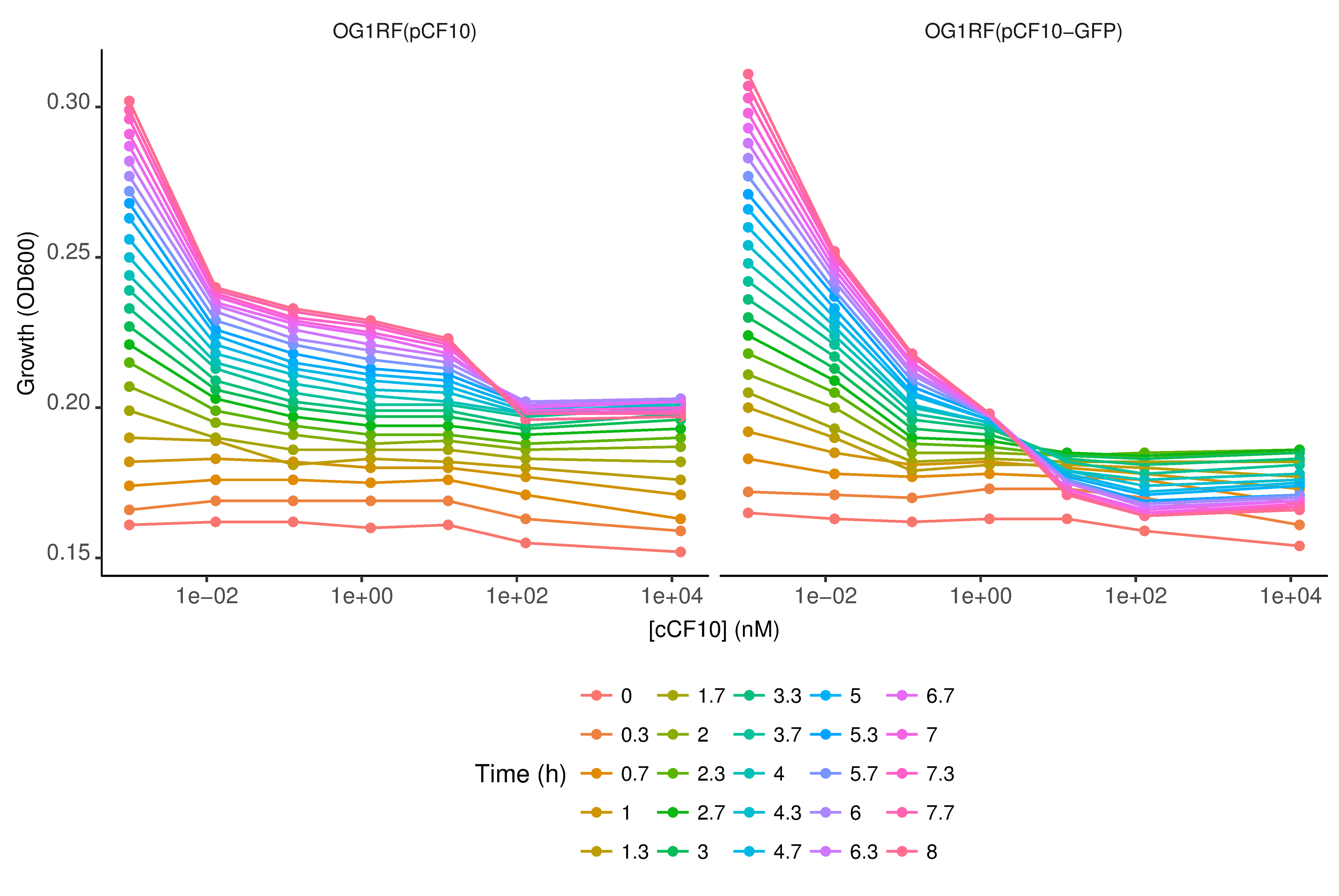

Supplement: S6 Fig — (A, B) Growth/adhesion assay showing mean growth (per well) of pCF10 (A, from Fig 3C) and pCF10-GFP (ΔprgU) (B). The underlying numerical data are shown in S12 Data. GFP, green fluorescent protein; Prg, pheromone responding gene. (TIF) [file pbio.3000814.s006.tif]

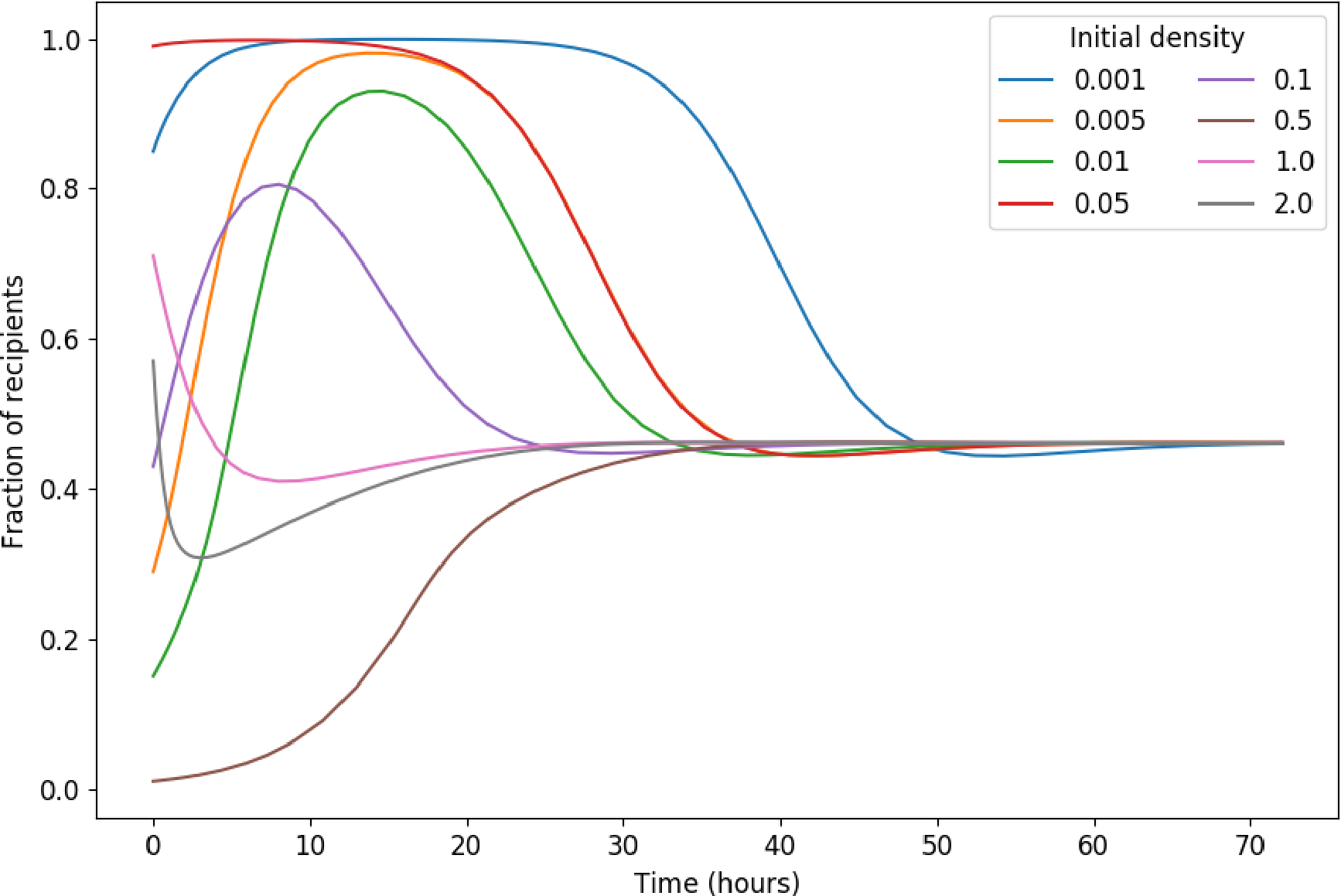

Supplement: S7 Fig — Representative example of a simulation showing the dynamics of (in this case) ratio-sensing strategy with different starting fraction of recipients and total population sizes. The underlying numerical data are shown in S13 Data. (TIF) [file pbio.3000814.s007.tif]

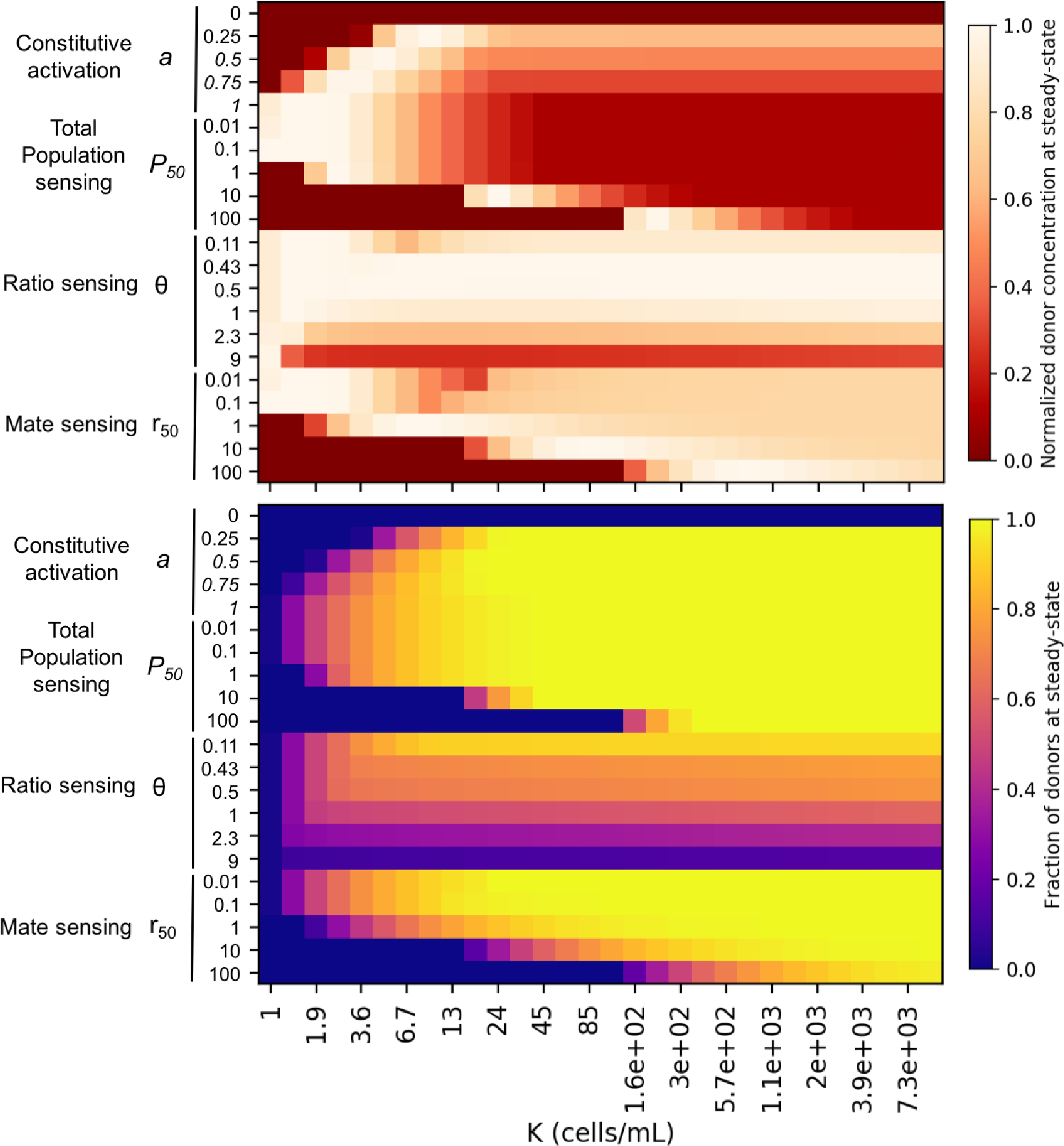

Supplement: S8 Fig — Ratio sensing maximizes donor fitness (top) and helps coexistence (bottom). Strategy comparison showing extended simulations varying relevant parameter values for each strategy: activation level (a) for the constitutive activation strategy and sensitivity (half-maximal activation; P50, θ, and r50; see Mathematical modeling) for the rest. Each value is normalized by the maximum value observed within each K. The underlying numerical data are shown in S14 Data. (TIF) [file pbio.3000814.s008.tif]
