# Supplementary material for: Ratiometric quorum sensing governs the trade-off between bacterial vertical and horizontal antibiotic resistance propagation
Source: PLoS Biol. 2020 Aug 14;18(8):e3000814. doi: 10.1371/journal.pbio.3000814 (PMC7449403; doi:10.1371/journal.pbio.3000814)
Supplement: Attachment — Submitted filename: Response to reviewers.pdf [file pbio.3000814.s023.pdf]

Response to reviewers

Response to Reviewer #1:

This manuscript focuses on defining the parameters and mechanism that allow plasmid-bearing antibiotic resistant cells to avoid being outcompeted in the absence of antibiotics. The authors evaluate several potential sensing mechanisms and conclude that ratiometric sensing of donor:recipient cells drives sexual behavior in bacteria. This is a well-executed study that report provocative results that should be of high interest to researchers studying sociomicrobiology and evolution. I also believe that the conclusions of this study are well-supported by the experimental data. I therefore recommend publication in PLOS Biology.

We thank Reviewer #1 for his/her positive evaluation of our work.

Response to Reviewer #2:

Banderas et al. present an interesting set of data on the function of the E. faecalis extracellular signaling system in the regulation of conjugation. A key finding is that the response changes a function of the composition of the population rather than by absolute cell density. This result is very convincingly demonstrated in Fig. 2. The authors also provide evidence that there is a trade-off between investing in horizontal versus vertical plasmid transfer as the induction of the pheromone pathway reduces the growth rate (Fig. 3). Finally, the authors present a model that could explain how ratio-sensing contributes to a stable equilibrium of donor-recipient ratio in the population based on a mathematical model (Fig. 4). I found the MS interesting and thought provoking and generally feel that it could qualify as a Short Communication.

We thank Reviewer 2 for the overall positive appreciation of our work.

However, I have a few remarks which should be considered.

Major comments

1. Ratio-sensing and signaling architecture: *The authors motivate their ratio-sensing hypothesis by highlighting specific features of regulatory architecture of the signaling system. In Figure 1 the authors propose that the ratio-sensing ability requires* ***a)*** *two signaling systems and they assume* ***b)*** *that the extracellular concentrations of both signals are proportional to the donor and recipient concentrations, respectively. These assumptions are not tested in the MS and they may not hold.*

It seems important to point out that the investigated signaling system functions by means of a peptide-based export-import circuit (a wide-spread signaling architecture found in many G+ bacteria, Neiditch et al., Annu. Rev. Genet. 2017. 51:311-33, https://doi.org/10.1146/annurev-genet-120116-023507). The authors do not mention/consider this aspect. However, this could be important to explain the phenomena observed by the authors. In a recent publication it was shown that export-import (or „pump-probe" signaling systems) extracellular concentrations do not necessarily increase with increasing population density. Moreover, such systems are capable of ratio-sensing in mixed populations of producer and non-producer cells, when signals are taken up very efficiently compared to overall signal production in the population (Babel et al., Nat. Commun. 11, 2020, https://doi.org/10.1038/s41467-020-14840-w). Hence, for ratio-sensing in E. faecalis conjugation, the second signaling system (assumption a) might in fact be dispensible and assumption b is by no means trivial and not necessarily to be expected. This should be considered/discussed.

Please either provide additional data to support their specific model or more carefully introduce/motivate/discuss the ratio-sensing hypothesis adopting a broader mechanistic perspective of the overall signaling architecture.

We focus here on the two critical aspects of the Reviewer's comments, our assumptions that **(A)** ratio-sensing requires a second signalling system (iCF10) and **(B)** *extracellular concentrations of both signals are proportional to the donor and recipient concentrations in perspective of newly published Babel 2020.* Both aspects are ultimately based on pheromone uptake (pumping) taking the role of a density-dependent sink for pheromones.

A. Ratio-sensing requires a second signalling system (iCF10)

The first concern is that iCF10 pheromone might be dispensable for ratiometric sensing in *E. faecalis.* In principle, yes. However, taking into account the following two aspects, we think that iCF10 remains essential :

I. It is necessary to point out that pCF10 plasmids with a non-functional version iCF10 are strong constitutive activators of the pathway. We show the phenotype here (See image below). This was reported before, although with no images available by Chatterjee et al (2013). This strain shows pathway saturation (autoaggregation) constitutively without external addition of cCF10, rendering any sensing capabilities impossible. Pheromone iCF10 keeps donors away from saturation by counteracting the inducing effect of "leaky" cCF10 production (Chatterjee et al 2013). As ratio sensing occurs in mixed populations, without iCF10 signal saturation is expected to be even more severe, as each recipient cell produces much more cCF10 than a donor (due to PrgY, new figure Fig 1C). In a general sense, these properties therefore render iCF10 indispensable for ratio sensing.

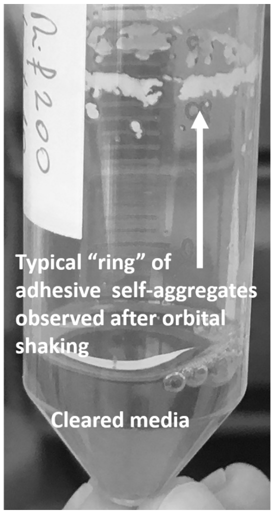

II. It is necessary to consider a difference between the requirements for ratiometric sensing in *Bacillus* (Babel et al, 2020 *Nature communications*, 11(1), 1-13; published after our submission) and the particularities of the pCF10 system.

In *Bacillus*, ratio sensing is aided by global signal uptake done by both emitter and receiver cells. Contrary to that, as *Enterococcus* plasmid-recipients simply secrete cCF10 as a waste product, pheromone internalization has evolved as donor-specific, with the PrgZ oppA-like protein being plasmid-encoded and specific for sex-pheromones. Therefore, global pheromone uptake is expected to be significantly weaker in recipient-biased populations, making pump saturation of donors (the ones that execute the output) more likely, arguing against the possibility of wide-range ratio-sensing purely by means of the pCF10 pump-probe system (Leonard et al, 1996, *PNAS* 93(1) 260-264; Berntsson et al 2012, *J Biol Chem*, 287(44), 37165-37170)

Considering (I) and (II), *we now discuss in detail the differences of the B. subtilis and E. faecalis systems. We conclude that even though we cannot rule out the effects of pumping in the ratiometric response, iCF10 remains a major player, and further suggest that both iCF10 and pumping could shape the observed ratio-sensing curve (See new discussion, lines 286-307).*

B. Extracellular concentrations of both signals are proportional to the donor and recipient concentrations in perspective of newly published Babel 2020.

The second concern is that extracellular pheromone concentrations might not necessarily increase with cell densities of their emitters. We fully acknowledge this fact, as we cannot rule out that signal depletion modifies extracellular pheromone concentrations. We agree with the Reviewer that we do assume that the respective signalling molecules are proportional to donors and recipients in our mixed populations. However we think this is still a reasonable assumption,

but one that requires more precision. Since our data demonstrates a direct relationship between cell ratio and donor response in mixed experiments, we did not focus on the details of iCF10 and cCF10 accumulation dynamics or steady state concentrations, as our evolutionary model rather only requires a phenomenological assessment of the relation (a transfer function) between population parameters and cell-responses. Therefore, we modify the text to give more precision to our language, considering the broader context of import-export systems.

First, we explicitly show signal internalization via the PrgZ/opp system, citing the relevant literature (new Fig. 1C; lines 70-71, 129-132) (including Neiditchet et al, 2017, Annual review of genetics, 51, 311-333 and the other two references above). We more carefully introduce the role of iCF10 to better motivate the hypothesis (lines 91-94), precising that it is rather the pheromones' accumulation rates are expected to be proportional their respective emitters, as opposed to (steady state) concentrations (line 99), and remove the mathematical expression for the new Fig. 1C, to avoid confusion.

In general, thanks to the Reviewer, we believe we are now better introducing and discussing the necessity of the iCF10 system in *E. faecalis* in the context of import-export systems. We discuss the pump-probe model (Babel et al 2020) in the specific context of the pCF10 system in *E faecalis* and *conclude that we cannot rule out that pumping contributes to the observed phenomenon.*

2. Stimulation experiments to support tradeoff model: *Again, given the network architecture, in the stimulation experiments performed in Fig. 3 it is not obvious that the extracellular concentration is indeed the relevant biophysical quantity as the signals will be imported by the cells and activate an intracellular receptor. It might be the available dose of signaling molecules not the extracellular concentrations of signaling molecules present at the start of the experiment, which matters. This point will likely not affect the conclusions of the experiment, since presumably cell densities were initially all the same in all experiments. Thus variation in concentrations are equivalent to variation in dose. Nevertheless, this point should at least be mentioned or alternatively clarified experimentally.*

We thank the Reviewer for raising this distinction. Indeed, densities were kept identical and thus the real pheromone "dose" is comparable across samples. We acknowledge this fact *by stating that reported concentrations are initial in the Results and Methods sections (lines 195-196, 349-350).*

3. Role of prgU/mathematical model: *Here I got confused how the tradeoff got linked to the model. Looks like the authors caveat against the tradeoff which they just demonstrated and then come up with an alternative hypothesis for the „function(?)" of ratio-sensing. Please clarify.*

We apologize for the lack of clarity of this section that now went through a major editing effort with a new model now incorporating the trade-off. Specifically, we have now included the cost of

the activation of the conjugation pathway in donor proliferation (new Eq. 1) and analyzed the individual fitness advantage that ratiometric sensing confers to plasmid fitness in environments of different nutrient availability. The model now clearly shows that ratio sensing simply allows the highest fitness for donors (plasmid spread), explaining its evolution (new Fig 4A, new Fig. S8). The result is stated in the Abstract (lines 34-36), Introduction (lines 139-143), Results (lines 267-273) and Discussion (lines 308-315):

Also consider to replace the title „Mathematical model for conjugation dynamics" by a statement that states the findings derived from the model.

As suggested, we replaced the title for “Ratio sensing increases plasmid fitness and facilitates donor-recipient coexistence".

I was also confused by the intended message, is the coexistence a „function" of ratio-sensing or a „consequence"?

We understand the confusion, and apologize for the incompleteness of the argument. As mentioned, the new model shows that the reason ratio sensing evolves is that it maximizes plasmid fitness. Additionally, compared to other strategies, ratio sensing has the highest capacity to allow coexistence (new figure Fig 4B, new Fig. S8). Such robust coexistence is therefore rather a “property” of ratio sensing, discussed in lines 312-315.

The discussion then brings up the antibiotics for this part. The presentation of this part is sub-optimal and needs improvement for clarity.

We no more mention antibiotics as a selection scheme for ratio sensing, as it is unnecessary given our new modelling results.

Minor comments

4. Check for typos in the text and the figures! e.g. Fig 1a.plamid/plasmid simulation/stimulation
We thank the Reviewer for spotting this!. We additionally found an error in the name of aggregation protein Asc10, which we have also corrected.

5. "monitoring the expression of a GFP reporter which is driven by a copy of prgB's ribosome binding site (RBS) further downstream in the transcript". Maybe reword to facilitate easier understanding.
We have replaced the cited paragraph with more precise wording (lines 134-136.

6. Fig. 2A/B. Please explain the color bar in the caption.

We reorganized the caption to include the explanation for color (lines 167-174).

7. Mathematical model: check consistency of notation for all parameters.
We have double checked all parameters for consistency.

8. „If 50 is however of an order of magnitude between those of (1 −/) and (1 -/), then the system behavior is not analysed here." Please explain a bit more.

Due to the improvements in the model, we have replaced the mathematical analysis paragraph (from which limited conclusions could be drawn) in favor of a supplementary figure (new Fig. S8), with extended simulations exploring the role of pathway sensitivity, strategy and resources on donor fitness. The figure shows that the highest generalized donor fitness is achieved with ratio sensing, when the sensitivity to ratio is close to our experimental observations.

Finally, we alert the Reviewer of an unintended mistake in the previous version of Figure 2C. We now change it to display the correct "activated donor fraction" rather than a wrongly calculated "activated donor ratio". The former is the one actually used for to estimate parameters theta and etha (methods), and therefore the correct one to show. Additionally,the x-scale is now shown with calculated logarithms rather than with a log scale, which caused irregular binning. We apologize for the mistake. Nevertheless, it does not alter the figure message and rather corrects an inconsistency in the previous version.

Response to Reviewer #3:

In this manuscript, Banderas et al. study the regulation of plasmid conjugation in the pathogenic bacterium Enterococcus faecalis. They show experimentally that conjugation is induced when the bacterial population is composed mostly of recipient (plasmid-free) cells, rather than when the overall population density is sufficiently high. Further experiments show that activation of conjugation is costly. The authors therefore argue that ratiometric control of conjugation mitigates this cost and ensures that donors only bear the cost of conjugation when recipients abund and the benefit of conjugation is high. Finally, the authors construct a mathematical model to study how the interplay between the costs and benefits of plasmid carriage affects the prevalence of plasmid-bearing cells in bacterial populations.

Overall, I find this work to be of high quality, novel and of broad interest. Ratiometric control of conjugation makes intuitive sense, and can significantly affect the dynamics of plasmid-born traits, such as pathogenicity and antibiotic resistance. Yet, I am not familiar with previous works discussing this mode of regulation and its implications.

We thank the Reviewer for the constructive review and the positive comments on the overall quality of our manuscript.

I do have major comments regarding the model as well as more minor ones, as detailed below.

Major comments

As formulated, the model is inadequate for its declared purpose - comparing conjugation regulation strategies. This is because the model only accounts for the constant cost of plasmid carriage and does not consider the cost of activating conjugation - a cost demonstrated in Fig. 3. Therefore, in this model activation of conjugation at any population density or composition can only be beneficial for plasmid spread. Indeed that is the case in the results shown in Fig. 4B, where ratio-sensing results in the lowest prevalence of donors. Moreover, I found the modeling section to be misleading, and at odds with the rest of the paper. The conclusion of this section is that ratio-sensing is "the only strategy allowing a robust co-existence of the two populations [donors and recipients]". Since in previous sections the authors argue that ratio-sensing may be optimal for the plasmid in mitigating the demonstrated cost of conjugation, I initially took that conclusion to mean that ratio sensing prevents the extinction of donor, thus allowing co-existence of recipient and donors. However, the model results in fact show the exact opposite - ratio sensing prevents the extinction of recipients. Since in this model ratio sensing is not beneficial to the plasmid which encodes the regulatory machinery implementing this regulation it is also not clear why such regulation would evolve. In the discussion, the authors argue that maintaining coexistence between carriers and plasmid-free cells may be beneficial to the population as a whole under intermittent antibiotic exposure. This is an interesting idea, and ratio sensing may be an evolutionary stable state under specific conditions. However, a simpler, and likely more robust mechanism for the evolution of ratio sensing is that it is directly beneficial to the plasmids that implement it. Therefore, the model needs to be revised to include a cost for conjugation. I realize this entails some arbitrary modeling decisions and the addition of parameters to the model. However, I believe that any reasonable choice can dramatically change the behavior of the model, and make it suitable to address the question of when is ratio sensing favorable to the donors. Since this manuscript is being considered as a Short Report, a simple "proof-of-concept" model showing the benefit of ratio sensing for plasmid donors would suffice. Such a model would likely motivate subsequent more comprehensive dedicated modeling efforts.

We agree with the Reviewer’s criticism and thank him/her for his/her suggestion and intuition that has significantly improved our model and its interpretation. The model, as it was presented in the previous submission, was indeed inadequate.

We have now included the cost of activating the conjugation pathway on the donor’s growth rate in accordance with our experimental observations. This is reflected in the model as a trade-off for activation (new Equation 1), with a maximum growth reduction of 20%, estimated from data from our growth assays (Fig S5). Our new simulations show that ratio sensing allows for the highest donor fitness compared to all other strategies (new Fig 4A and S8, lines 240-277), suggesting its evolutionary selective advantage. Importantly, robust stable-coexistence remains an important conclusion of our simulations. The new model shows

that when there is low carrying capacity (*K*), ratio sensing allows donors to resist recipient take-over, as suspected by the Reviewer (new Fig 4B). When resources are abundant, other strategies allow donors to take over, however, despite maintaining recipients in the population, ratio sensing shows higher absolute donor densities (Fig 4A and S8). Robust coexistence is discussed now as an efficient way to increase plasmid fitness in times of no antibiotic exposure (lines 308-315).

Additional comments

**The experiments demonstrating the cost of conjugation do not rule out the possibility of cCF10 toxicity. The claim would be significantly strengthened by additional experiments showing that plasmid-free recipient strains are unaffected by exposure to the same concentrations of cCF10.*

We agree with the Reviewer. This is a simple control to perform, however difficult because of lockdown-related restrictions. To derive our conclusions we focus on the physiological range of cCF10 sensing, namely concentrations below pathway saturation (~1 nM, new Fig. 3, new Fig. S5), as it is the relevant range for ratio sensing and mating (Fig 2). That said, there are a few facts we believe reduce the significance of the suggested experiment:

1. First, It has been demonstrated that cCF10-mediated toxicity indeed exists. However, it is *prgB* mediated and therefore exclusive to Donors (Bhatty et al 2017, reference in text)
2. Second, cCF10 import (required for the toxic effects) requires prgZ, also a plasmid-encoded protein, and therefore absent in recipients (new references Leonard et al 1996 *PNAS*, 93(1), 260-264; Berntsson et al 2012, J Bio Chem, 287(44), 37165-37170)
3. During their normal growth in monocultures, recipients normally constitutively produce cCF10 (technically, a chopped-off signal peptide) at concentrations high enough to fully stimulate donors (Fig. 2): This makes non prgB-mediated toxicity highly unlikely, as high production could be easily selected out by evolution.
4. In donor-recipient mixtures, any possible plasmid-independent toxic effect of any pheromone is equal for both cell types, making it rather a “background effect”

We now explicitly mention that fitness reduction is likely to be related to prgB induced toxicity, not observed previously for the wild type strain but only for the sensitized *prgU* knockout, which we also confirm here (lines 234-238).

**I find it unlikely that conjugation regulation is insensitive to population density even at very low population densities, as stated in the text and implied in Fig. 1B. At low population densities, it would be unlikely for a donor to encounter a recipient even at a high R:D ratio. The current experiments demonstrating ratiometric regulation are all done in very high population densities (OD 0.1-1), therefore it is still unknown how conjugation is regulated at low population densities. I am not advocating that the authors conduct further experiments, since the novelty is in the fact that ratiometric regulation occurs at all. But, this caveat should be stated and discussed.*

We agree with the comment. We do not expect ratiometric sensing at very low densities. Experimentally, this would mean working at densities that correspond to concentration under 10 pM, well under any capacity of our reporter system. We now discuss that ratio sensing is unlikely at low densities, for the reasons suggested by the Reviewer (lines 281-285).

**More information is required regarding the experiments shown in Fig. 3B. Data from how many replicates is shown? Or is it a single replicate per condition?*

This data is a representative experiment. We now added additional replicates at the relevant physiological (sub-nanomolar) cCF10 concentrations (newly added Fig. S5).

**Additionally, the histograms to the right have some white dots in them. Is that an issue with the rendering?*

We assume the Reviewer means the left side. It is indeed the rendering. We have simplified visualization to clearly show that the data is organized as discrete points. To reduce the point density, we now show only a horizontal section of the microwell scan, with essentially the same result but a smoother and clearer visualization (New Fig. 3B)

**I would replace Fig. 4A, with a heatmap showing the steady-state donor fraction as a function of initial total population size and donor fraction.*

We are unsure of the figure intended in this comment since all initial conditions lead to the same donor fraction at steady state, such heatmap would be of a uniform color. Therefore we believe our presentation to be more telling (trajectories). Taking in consideration the Reviewer's suggestions on the model, we now moved the trajectories to new Figure S7, as the main conclusion deals with donor fitness and stability robustness, rather than stability itself. Nevertheless, heatmaps are included to show the dependence of donor fitness on strategies, sensitivity, and carrying capacity (new Figure S8)

**While arbitrary, the choice of nM units for the total population size 'K' in panel B is unusual and confusing. Suggest replacing it with cells/ml.*

We agree. We have performed the suggested change.

Finally, we alert the Reviewer of an unintended mistake in the previous version of Figure 2C. We now change it to display the correct "activated donor fraction" rather than a wrongly calculated "activated donor ratio". The former is the one actually used for to estimate parameters theta and etha (methods), and therefore the correct one to show. Additionally,the x-scale is now shown with calculated logarithms rather than with a log scale, which caused irregular binning. We apologize for the mistake. Nevertheless, it does not alter the figure message and rather corrects an inconsistency in the previous version.
