# Supplementary material for: Ratiometric quorum sensing governs the trade-off between bacterial vertical and horizontal antibiotic resistance propagation
Source: PLoS Biol. 2020 Aug 14;18(8):e3000814. doi: 10.1371/journal.pbio.3000814 (PMC7449403; doi:10.1371/journal.pbio.3000814)
Supplement: Attachment — Submitted filename: Response to reviewers.docx [file pbio.3000814.s024.docx]

We thank the Reviewers for the positive evaluation of the revised version of our Manuscript

Kindly yours,

Alvaro Banderas and Ariel B. Lindner
